# Supplementary material for: Spatial and Temporal Dynamics of Chikungunya Incidence in Brazil and the Impact of Social Vulnerability: A Population-Based and Ecological Study
Source: Diseases. 2024 Jun 27;12(7):135. doi: 10.3390/diseases12070135 (PMC11276368; doi:10.3390/diseases12070135)
Supplement: Supplementary file 1 [file diseases-12-00135-s001.zip › diseases-2981271-supplementary.pdf]

Spearman's Correlations

Variable		INCP1	IVS	IVSIF	IVSCH	IVSRT	A	B	C	J	L	M	O	S	T	U	V	Z	FF	JJ	MM	NN	QQ	SSS	TTT	VVV	ZZZ
1. INCP1	Spearman's rho	—																									
	p-value	—																									
2. SVI	Spearman's rho	0.348	—																								
	p-value	< .001	—																								
3. SVIID	Spearman's rho	0.278	0.854	—																							
	p-value	< .001	< .001	—																							
4. SVIHCD	Spearman's rho	0.347	0.930	0.665	—																						
	p-value	< .001	< .001	< .001	—																						
5. SVIIWD	Spearman's rho	0.341	0.910	0.616	0.867	—																					
	p-value	< .001	< .001	< .001	< .001	—																					
6. A	Spearman's rho	0.355	0.797	0.701	0.735	0.714	—																				
	p-value	< .001	< .001	< .001	< .001	< .001	—																				
7. B	Spearman's rho	0.291	0.744	0.657	0.670	0.680	0.632	—																			
	p-value	< .001	< .001	< .001	< .001	< .001	< .001	—																			
8. C	Spearman's rho	0.044	0.376	0.669	0.185	0.119	0.141	0.124	—																		
	p-value	0.001	< .001	< .001	< .001	< .001	< .001	< .001	—																		
9. J	Spearman's rho	0.381	0.839	0.580	0.861	0.854	0.684	0.611	0.099	—																	
	p-value	< .001	< .001	< .001	< .001	< .001	< .001	< .001	< .001	—																	
10. L	Spearman's rho	0.217	0.788	0.502	0.848	0.814	0.567	0.577	0.057	0.776	—																
	p-value	< .001	< .001	< .001	< .001	< .001	< .001	< .001	< .001	< .001	—																
11. M	Spearman's rho	0.360	0.831	0.607	0.857	0.793	0.700	0.589	0.190	0.796	0.655	—															
	p-value	< .001	< .001	< .001	< .001	< .001	< .001	< .001	< .001	< .001	< .001	—															
12. O	Spearman's rho	0.295	0.426	0.316	0.395	0.448	0.250	0.241	0.269	0.257	0.254	0.464	—														
	p-value	< .001	< .001	< .001	< .001	< .001	< .001	< .001	< .001	< .001	< .001	< .001	—														
13. S	Spearman's rho	-0.283	-0.894	-0.612	-0.923	-0.904	-0.692	-0.669	-0.108	-0.858	-0.933	-0.783	-0.328	—													
	p-value	< .001	< .001	< .001	< .001	< .001	< .001	< .001	< .001	< .001	< .001	< .001	< .001	—													
14. T	Spearman's rho	-0.313	-0.845	-0.589	-0.858	-0.857	-0.645	-0.622	-0.124	-0.805	-0.816	-0.728	-0.410	0.913	—												
	p-value	< .001	< .001	< .001	< .001	< .001	< .001	< .001	< .001	< .001	< .001	< .001	< .001	< .001	—												
15. U	Spearman's rho	-0.226	-0.826	-0.545	-0.876	-0.839	-0.609	-0.619	-0.083	-0.772	-0.955	-0.690	-0.303	0.970	0.841	—											
	p-value	< .001	< .001	< .001	< .001	< .001	< .001	< .001	< .001	< .001	< .001	< .001	< .001	< .001	< .001	—											
16. V	Spearman's rho	-0.331	-0.886	-0.635	-0.877	-0.898	-0.725	-0.662	-0.119	-0.904	-0.801	-0.833	-0.276	0.923	0.843	0.822	—										
	p-value	< .001	< .001	< .001	< .001	< .001	< .001	< .001	< .001	< .001	< .001	< .001	< .001	< .001	< .001	< .001	—										
17. Z	Spearman's rho	-0.145	-0.740	-0.455	-0.778	-0.798	-0.527	-0.546	-0.013	-0.771	-0.943	-0.615	-0.151	0.913	0.779	0.932	0.804	—									
	p-value	< .001	< .001	< .001	< .001	< .001	< .001	< .001	0.349	< .001	< .001	< .001	< .001	< .001	< .001	< .001	< .001	—									
18. FF	Spearman's rho	-0.331	-0.886	-0.635	-0.877	-0.898	-0.725	-0.662	-0.119	-0.904	-0.801	-0.833	-0.276	0.923	0.843	0.822	1.000	0.804	—								
	p-value	< .001	< .001	< .001	< .001	< .001	< .001	< .001	< .001	< .001	< .001	< .001	< .001	< .001	< .001	< .001	< .001	< .001	—								
19. JJ	Spearman's rho	0.292	0.884	0.650	0.890	0.860	0.715	0.665	0.163	0.810	0.794	0.776	0.339	-0.883	-0.826	-0.826	-0.865	-0.745	-0.865	—							
	p-value	< .001	< .001	< .001	< .001	< .001	< .001	< .001	< .001	< .001	< .001	< .001	< .001	< .001	< .001	< .001	< .001	< .001	< .001	—							
20. MM	Spearman's rho	-0.144	-0.422	-0.401	-0.446	-0.276	-0.353	-0.331	-0.230	-0.176	-0.306	-0.308	-0.359	0.370	0.379	0.375	0.270	0.178	0.270	-0.379	—						
	p-value	< .001	< .001	< .001	< .001	< .001	< .001	< .001	< .001	< .001	< .001	< .001	< .001	< .001	< .001	< .001	< .001	< .001	< .001	< .001	—						
21. NN	Spearman's rho	0.451	0.537	0.499	0.504	0.453	0.468	0.440	0.283	0.344	0.324	0.443	0.511	-0.396	-0.440	-0.360	-0.368	-0.171	-0.368	0.440	-0.473	—					
	p-value	< .001	< .001	< .001	< .001	< .001	< .001	< .001	< .001	< .001	< .001	< .001	< .001	< .001	< .001	< .001	< .001	< .001	< .001	< .001	< .001	—					
22. QQ	Spearman's rho	0.455	0.555	0.496	0.509	0.504	0.462	0.449	0.268	0.380	0.345	0.456	0.523	-0.420	-0.466	-0.381	-0.400	-0.206	-0.399	0.464	-0.378	0.968	—				
	p-value	< .001	< .001	< .001	< .001	< .001	< .001	< .001	< .001	< .001	< .001	< .001	< .001	< .001	< .001	< .001	< .001	< .001	< .001	< .001	< .001	< .001	—				
23. SSS	Spearman's rho	0.353	0.833	0.671	0.833	0.747	0.662	0.595	0.257	0.666	0.670	0.715	0.497	-0.777	-0.774	-0.727	-0.736	-0.562	-0.736	0.832	-0.611	0.624	0.623	—			

Spearman's Correlations

Variable		INCP1	IVS	IVSIF	IVSCH	IVSRT	A	B	C	J	L	M	O	S	T	U	V	Z	FF	JJ	MM	NN	QQ	SSS	TTT	VVV	ZZZ
	p-value	< .001	< .001	< .001	< .001	< .001	< .001	< .001	< .001	< .001	< .001	< .001	< .001	< .001	< .001	< .001	< .001	< .001	< .001	< .001	< .001	< .001	< .001	—			
24. TTT	Spearman's rho	0.380	0.842	0.583	0.863	0.856	0.687	0.614	0.101	1.000	0.778	0.797	0.258	-0.860	-0.808	-0.774	-0.906	-0.772	-0.906	0.815	-0.182	0.346	0.381	0.670	—		
	p-value	< .001	< .001	< .001	< .001	< .001	< .001	< .001	< .001	< .001	< .001	< .001	< .001	< .001	< .001	< .001	< .001	< .001	< .001	< .001	< .001	< .001	< .001	< .001	—		
25. VVV	Spearman's rho	-0.334	-0.893	-0.647	-0.874	-0.906	-0.769	-0.703	-0.117	-0.839	-0.781	-0.812	-0.299	0.882	0.820	0.807	0.902	0.746	0.902	-0.876	0.334	-0.463	-0.482	-0.769	-0.842	—	
	p-value	< .001	< .001	< .001	< .001	< .001	< .001	< .001	< .001	< .001	< .001	< .001	< .001	< .001	< .001	< .001	< .001	< .001	< .001	< .001	< .001	< .001	< .001	< .001	< .001	—	
26. ZZZ	Spearman's rho	0.244	0.552	0.421	0.540	0.539	0.481	0.461	0.135	0.409	0.439	0.495	0.358	-0.465	-0.479	-0.470	-0.359	-0.336	-0.359	0.557	-0.375	0.531	0.526	0.567	0.411	-0.611	—
	p-value	< .001	< .001	< .001	< .001	< .001	< .001	< .001	< .001	< .001	< .001	< .001	< .001	< .001	< .001	< .001	< .001	< .001	< .001	< .001	< .001	< .001	< .001	< .001	< .001	< .001	—

Legenda:

INCP1: Incidence of Chikungunya in Brazil.
SVI: Social Vulnerability Index.
SVIID: Social Vulnerability Index - Infrastructure Dimension
SVIHCD: Social vulnerability index – human capital dimension
SVIIWD: Social vulnerability index – income and work dimensions.
A: Percentage of people in households with inadequate water supply and sanitation.
B: % of the population living in urban households without garbage collection services.
C: % of people who live in households with a per capita income of less than half the minimum wage (as of 2010) and who spend more than an hour commuting to work.
J: Illiteracy rate of the population aged 15 and over.
L: Percentage of children living in households where none of the residents have completed primary education.
M: Percentage of people aged 15 to 24 who do not study, do not work and have a per capita household income equal to or less than half the minimum wage (as of 2010).
O: Unemployment rate of the population aged 18 and over.
S: Municipal human development index (IDHM)
T: IDHM Longevity.
U: IDHM Education.
V: IDHM Income.
Z: Education subindex - IDHM Education.
FF: Per capita income.
JJ: Dependency ratio.
MM: Aging rate.
NN: Vulnerable population aged 15 to 24.
QQ: Population in vulnerable households and with elderly people.
SSS: Percentage of population in households with density > 2.
TTT: Illiteracy rate - 18 years and over.
VVV: Per capita income of those vulnerable to poverty.
ZZZ: Gini index.

Results of the descriptive analysis

The analyses revealed a total of 487.775 cases of Chikungunya diagnosed in Brazil between 2017 and 2023, with an incidence rate of 232.1 per 100.000 inhabitants. The most commonly used diagnostic criterion was clinical-epidemiological. Regarding the epidemiological characteristics of the population, the highest rates were observed in the Northeast region (58.8 per 100.000 inhabitants), among the female gender (61.8 per 100.000 inhabitants), individuals of mixed race (75.3 per 100.000 inhabitants), and the age group of 60 to 69 years (34.7 per 100.000 inhabitants).

Supplementary Table. Sociodemographic characteristics and incidence rate of Chikungunya in Brazil, 2017 – 2023

Variable/Category	Chikungunya		
	n	%	Incidence
Brazil	487.775	100.0	232.1
Region of residence			
North	28.678	6.0	15.5
Northeast	283.075	58.0	49.3
Southeast	152.929	31.3	17.1
Central-West	21.594	4.4	13.0
South	1.449	0.30	4.8
Gender			
Female	301.447	61.8	27.8
Male	186.278	38.2	17.9
Race			
White	84.895	17.4	45.4
Black	27.050	5.5	38.8
Yellow	7.260	1.5	14.4
Brown	367.024	75.3	196.5
Indigenous	1.496	0.30	8.0
Age Group			
< 1 ano	5.072	1.0	3.4
1 – 4	8.388	1.7	5.7
5 – 9	17.920	3.6	12.2
10 – 14	25.359	5.2	17.1

15 – 19	30.968	6.3	196.1
20 – 39	169.598	34.7	99.8
40 – 59	151.953	31.1	97.3
60 – 64	25.766	5.3	27.4
65 – 69	20.044	4.1	27.2
70 – 79	23.364	5.0	43.1
80+	9.293	2.0	39.2
Diagnostic criterion			
Laboratory	186.761	38.3	88.1
Clinical-epidemiological	300.964	61.7	142.1
